# Plasma Chymase Activity Reflects the Change in Hemodynamics Observed after the Surgical Treatment of Patent Ductus Arteriosus in Dogs

**DOI:** 10.3390/vetsci9120682

**Published:** 2022-12-08

**Authors:** Kazumi Shimada, Lina Hamabe, Miki Hirose, Momoko Watanabe, Aimi Yokoi, Aki Takeuchi, Yusuke Ozai, Tomohiko Yoshida, Shinji Takai, Denan Jin, Meric Kocaturk, Katsumi Uehara, Ryou Tanaka

**Affiliations:** 1 Department of Veterinary Surgery, Tokyo University of Agriculture and Technology, 3-5-8 Saiwai-cho, Fuchu 183-8509, Japan; 2 Department of Innovative Medicine, Graduate School of Medicine, Osaka Medical and Pharmaceutical University, Takatsuki-City 569-8686, Japan; 3 Department of Internal Medicine, Faculty of Veterinary Medicine, Bursa Uludag University, Bursa 16059, Turkey; 4 Masamitsu Co., Ltd., Saitama 369-0305, Japan

**Keywords:** chymase, echocardiography, patent ductus arteriosus

## Abstract

**Simple Summary:**

Chymase is a type of protease associated with tissue injury, inflammation, and the remodeling of the cardiovascular system. Chymase has been suspected to increase with the progression of cardiovascular diseases. The measurement of chymase activity can only be taken at the time of cardiovascular failure and requires tissue sampling. Therefore, chymase has not been examined in both human and veterinary medicine. In the present study, chymase activity was measured by using plasma from dogs diagnosed with patent ductus arteriosus, a commonly observed congenital heart disease condition in dogs. Moreover, as patent ductus arteriosus can be treated surgically, chymase activity was also measured in the post-operation plasma. The results of the present study showed that chymase activity could be measured from plasma in dogs with patent ductus arteriosus, and a decreased chymase activity was observed with an improvement in hemodynamics due to the surgical treatment. These new findings provide important information about the chymase mechanism in the field of veterinary medicine. In the future, chymase activity may be necessary for routine examinations of cardiac disorders in veterinary medicine.

**Abstract:**

Chymase is a protease stored in mast cell granules that produces angiotensin II (ANG II) from angiotensin I (ANG I) and is associated with tissue injury, inflammation, and remodeling, especially involving the cardiovascular system. As cardiovascular events occur, chymase is activated by degranulation to the extracellular matrix. Although chymase has been suggested to be associated with cardiovascular disease progression, there are not enough reports in veterinary medicine. Patent ductus arteriosus (PDA) is a common congenital cardiac disease in veterinary medicine. Almost all cases of PDA can be treated surgically to prevent the development of congestive heart disease and/or pulmonary hypertension. The aims of the present study were to measure chymase activity before and after PDA occlusions, and to investigate the relationships between the congestive and hemodynamic states of PDA and chymase activity. In the present study, 17 puppies diagnosed with PDA were included and all puppies completely recovered to the level of healthy dogs. Chymase activity significantly decreased at 2 months after the operation, along with the echocardiography parameters of congestion. Therefore, plasma chymase activity may be useful as a novel predictor for understanding the hemodynamics of PDA in veterinary medicine.

## 1. Introduction

Chymase is found in mast cells with a broad tissue and species expression [1]. Although chymase is stored in mast cell granules as a fully active form, it has no functional effect. Chymase is activated after the mast cells degranulate to the extracellular matrix in response to the presence of inflammatory cytokines or a tissue injury [2]. Angiotensin I (ANG I) is converted to angiotensin II (ANG II) by the active chymase as well as angiotensin-converting enzyme (ACE). In cardiac tissue, ANG II generation is mediated mainly by ACE [3]. ACE-derived ANG II is responsible for blood pressure regulation as well as the water and electrolyte balance through the renin–angiotensin–aldosterone system (RAAS). On the other hand, chymase is suggested to have the important role of contributing to tissue remodeling and cardiovascular disease (CVD) progression [2,4]. Most reports of chymase have been targeted to human diseases such as myocardial infarctions and arterial diseases [4]. There are few reports and insufficient information on chymase in congenital CVD. Although studies on experimental animals have been reported, the function of chymase in clinical animals is still unknown in veterinary medicine.

Patent ductus arteriosus (PDA) is a well-known congenital CVD. The ductus arteriosus (DA) is important for systemic circulation in the fetus until the time of birth. After birth, the closure of the duct spontaneously occurs in a normal heart [5]. The prostaglandin pathway is recognized as a valuable pharmacological target for maintaining the DA patency [6,7]. In human medicine, cyclooxygenase (COX) inhibitors are regarded as the mainstay therapy for PDA closure in premature infants [8], and the administration of indomethacin is commonly used for the treatment of PDA at 1–2 days of age. Untreated PDA progresses to heart failure and results in several complications such as pulmonary edemas and pulmonary hypertension [9]. However, in human medicine, it is known that the treatment of PDA may close or remain patent, depending on various clinical conditions such as the presence of other heart malformations [10]. In veterinary medicine, the medical treatment of PDA at 1–2 days after birth is not feasible; therefore, veterinarians usually decide on a surgical treatment for PDA to avoid heart failure progression [11,12,13,14]. In puppies, a surgical treatment such as a PDA occlusion decreases fractional shortening (FS) [15], which induces the dysfunction of the left arterial systolic function [16], a poor physical condition, and, temporarily, the presence of hypertension [17]. Moreover, after an occlusion of PDA, mitral valve regurgitation (MR) occasionally appears resulting from a volume overload of the left ventricle. Although echocardiography parameters [18,19] and clinical biomarkers [20,21] have been used to predict the outcomes of PDA in both human and veterinary medicine, no practical indicators have yet been reported in a clinical study.

Chymase was shown to have a critical role in myocardial wall thinning and LV chamber dilation in a pure volume overload in dogs with experimentally produced MR [4,22]. In addition, hemodynamic factors (such as increased wall stress) were reported to be important stimuli for chymase production/activation [4,23]. Therefore, the present study focused on chymase activity as a novel parameter to evaluate the changes in hemodynamics after the closure of PDA, which is a volume overload disease.

The measurement of chymase has issues. Chymase is mediated by an acute tissue injury and chronic remodeling [4], and chymase is stored in tissues, mainly in mast cells. The most accurate method requires tissue sampling. To measure the plasma chymase concentration, we know that immunobiochemical methods such as Western blotting are known to reflect the quantification of chymase protein more accurately. However, the sampling of tissues, especially cardiac and vascular tissues, is impossible to perform as a routine clinical practice [24]. Additionally, these methods are cumbersome and time-consuming. Therefore, the measurement of chymase, which may be important in CVD, needs to be convenient and non-invasive. A blood sample is the most appropriate method to measure chymase in a clinical setting because CVD patients commonly take blood tests. However, there are numerous strong factors that inhibit plasma chymase [25], which makes the measurement of plasma chymase more difficult than tissue chymase. Although the measurement of blood chymase in CVD patients is extremely difficult, an innovative method was reported that allowed the measurement of plasma chymase activity from hypertension patients [1]. In this report by Wang et al., a biochemical method was used to evaluate plasma chymase activity as the amount of chymase protein. Human chymase is similar to dog chymase in its structure, reaction, and function; the Suc-Val-Ala-Ala-Pro-Phe-pNA sequence used in the report by Caughey et al. can be used to measure chymase activity in both humans and dogs [26]. Therefore, this novel method to measure chymase activity could be extrapolated to veterinary medicine.

The aims of the present study were to measure chymase activity before and after the surgical treatment of PDA in dogs, and to investigate the relationships between the congestive and hemodynamic states of PDA and chymase activity.

## 2. Materials and Methods

### 2.1. Animals

Puppies were brought to the Dog and Cat Pediatric Hospital for the investigation of cardiac murmurs. Puppies were diagnosed with PDA with echocardiography. As the conventional echocardiographic parameters, the diameter of PDA was measured and fractional shortening (FS%) was calculated using the Pombo method: left ventricular internal diameter (LVID) at the end-diastole-LVID at the end-systole/LVID at the end-systole × 100. The early diastolic left ventricular inflow velocity (E vel) was measured using a pulse-wave Doppler; the diastolic early mitral ring velocity (e’) at the interventricular septum (IVS) and left ventricular free wall (LVFW) were measured using a pulse-wave tissue Doppler. The echocardiographic examination was performed using a Lisendo 880LE (FUJIFILM, Tachikawa, Japan) with a 14-3 MHz phased array transducer probe (S42; FUJIFILM, Japan).

A few cases required treatments with diuretics, ACE inhibitors, pimobendane, and antibiotics because they showed signs of congestion and needed to maintain a clinical condition prior to the operation. All treatments began after the echocardiographic examination and blood sampling.

### 2.2. Surgical Occlusion of PDA

Puppies were premedicated with buprenorphine (50 μg/kg) and midazolam (0.2 mg/kg), and anesthesia was induced with propofol (6 mg/kg to effect) from a venous catheter (24 or 26 G). Atropine (25 μg/kg) was injected when the heart rate fell below 100 beats per minute. All puppies were treated with an intravenous prophylactic antibiotic of cefazolin (20 mg/kg). After intubation, anesthesia was maintained by isoflurane (1–2%) with 100% oxygen and ventilation. Suxamethonium chloride hydrate (0.2 mg/kg for a single shot) was injected as a muscle relaxant. Shaving and disinfection were performed before draping. A thoracotomy at the left fourth intercostal space was performed and the ductus arteriosus was occluded using a vascular clip (AESCULAP^®^ Ligation Clips, M single or ML double size). Additionally, transesophageal echocardiography was performed for cases weighing over 850 g to confirm the complete occlusion of the ductus. The chest was closed as per the standard methods. The iatrogenic pneumothorax was corrected using a thoracic drain, which was removed on the following day after the operation in all cases. As a post-operative analgesia, buprenorphine (0.02 mg/kg) and/or bupivacaine (0.5 mg/kg) as a local anesthesia was administrated, depending on the degree of pain. After the operations, all cases were cared for in an O_2_ room (40%) until the removal of the thoracic drain. Cardiac medications were administrated through an intravenous catheter. By the point of discharge from the hospital, no cases required any cardiac medication.

The outcome of the operation was evaluated by echocardiography at one month post-operation. The cases with an incomplete PDA occlusion were excluded from the study.

### 2.3. Plasma Sample and Measurement of Plasma Chymase Activity

After the diagnosis of PDA, blood was sampled from a vein. Plasma was extracted from the blood with heparin by centrifugation (4000 rpm, 5 min). The excess plasma was frozen at −80 °C. Two months after the PDA occlusion, echocardiography and blood tests were performed. For chymase activity measurements, the excess plasma was stored in a freezer. Chymase activity was measured according to the method of a previous study [1]. A total of 50 μL of dissolved and centrifuged plasma was mixed with an equal volume of Tris buffer (100 mM Tris and 1 M NaCl at pH 8.0) and 1 μL of DMSO was added to one sample well and 1 μL of the chymase inhibitor (10 mM Suc-Val-Pro-Phe^p^ (0 Ph)_2_) was added to the other well. After one hour at room temperature, 1 μL of the chymase substrate (50 mM Suc-Val-Ala-Ala-Pro-Phe-pNA in DMSO) was added and mixed using a plate mixer for 5 min at room temperature. After 1 and 2 h of incubation at 37 °C, the plate was read at 405 mm with a microplate reader. The unit of chymase activity, U, was delivered as μmol/min pNA.

### 2.4. Statistical Analysis

The age at diagnosis and operation were counted as days from birth and presented as a median (minimum–maximum). The body weight, echocardiographic parameters, and chymase activity were presented as the mean ± standard deviation (SD). A non-parametric Wilcoxon test was used for chymase activity and the echocardiography parameters (GraphPad Prism 8). A *p*-value < 0.05 was considered to be of statistical significance.

## 3. Results

### 3.1. Animals and Echocardiography

**Figure 1:**
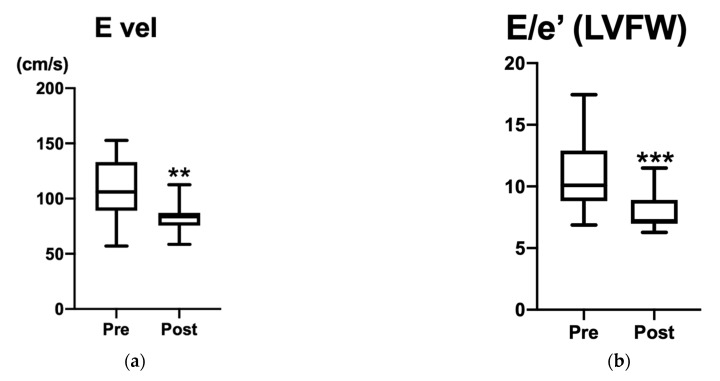
Echocardiography parameters: (**a**) E vel (cm/s) and (**b**) E/e’ at LVFW. E velocity is shown in (**a**) and E/e’ is shown in (**b**). Significant differences are indicated by ** (*p* = 0.0024) and *** (*p* = 0.0008). FS and E/e’ at septal were not significantly different between Pre and Post. Improvement in congestion was observed in all cases following PDA occlusion.

A total of 17 puppies, including 11 Pomeranians, 2 Toy Poodles, 2 Toy mixed-breeds, 1 Maltese, and 1 Bichon Frise, were diagnosed as PDA and the evaluation of chymase activity was performed. There were eight males and nine females. At the time of the initial presentation, they were 60 (59–73) days of age and weighed 0.59 ± 0.23 kg. At the time of the operation, they were 70 (62–117) days of age and weighed 0.72 ± 0.19 kg. The echocardiographic parameters are shown in Table 1. At the time of the echocardiographic evaluations, the diameter of PDA was 1.74 ± 0.58 mm and the FS was 39.4 ± 11.8%. The E vel was 108.3 ± 27.2 cm/s and the early diastolic mitral ring velocity (E/e’) at the IVS and LVFW were 12.2 ± 3.8 and 10.9 ± 2.9, respectively. Two months after the operations, the FS increased to 40.8 ± 8.0%, the E vel decreased to 82.9 ± 12.8 cm/s, and the E/e’ at the IVS and LVFW decreased to 9.7 ± 2.1 and 8.0 ± 1.6, respectively. The E vel and E/e’ at the LVFW significantly decreased (*p* = 0.0024 and *p* = 0.0008, respectively) (Figure 1). The changes in the parameters were suggestive of an improvement in the left ventricular congestion. All puppies recovered to the level of healthy dogs with an echocardiographic evaluation without cardiac medication.

### 3.2. Plasma Chymase Activity

**Figure 2:**
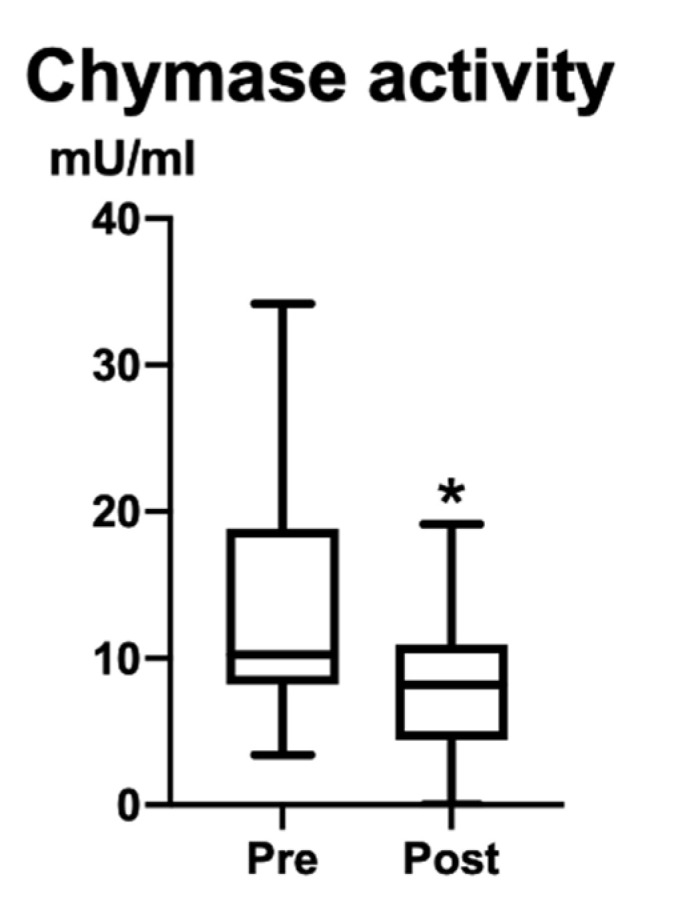
Plasma chymase activity (mU/mL). Significant difference is indicated by * (*p* = 0.049; 95% confidence interval −11.63 to 0.0000). After operations (Post), chymase activity significantly decreased.

Plasma chymase activity before the operations (Pre) was 13.4 ± 8.8 U/mL; after two months (Post), it decreased to 8.3 ± 4.7 U/mL. A significant difference appeared between Pre and Post (*p* = 0.049; 95% confidence interval −11.63 to 0.0000) (Figure 2).

## 4. Discussion

In veterinary medicine, the treatment of choice for PDA is a surgical occlusion. The reasons are that many puppies are diagnosed with PDA after passing time from birth, and an occlusion using indomethacin may not be effective. In the present study, all puppies were approximately two months old and the surgical occlusion was performed early because they had a large ductal size for their body size. Seventeen puppies were re-examined two months after their operations and an echocardiographic examination revealed that the cardiac function had recovered to the level of healthy dogs.

Chymase is difficult to measure as a routine practical parameter because it is not measured directly from circulating blood [24]. Therefore, only a few clinical studies have been reported in both human and veterinary medicine. However, the measurement of chymase activity from a blood sample is desired as CVD cases have increased risks of anesthesia, and cardiac and vascular tissue samples can only be obtained invasively. In the present study, chymase activity was successfully measured from blood samples, based on the chymase protein reacting by using a biochemical method. This novel method was useful, and proved to be non-invasive for puppies weighing under 1 kg diagnosed with PDA. This result indicated that plasma chymase activity might meet the requirements of becoming a novel parameter for PDA. Many reports have investigated serum biochemical markers as indicators of hemodynamic-significant PDA, both independently and with echocardiography [27]. Although comparisons with these markers are needed, this measurement of chymase activity may be useful for veterinary medicine, especially dogs.

Chymase activity showed a significant reduction after the operations. After the operations, PDA occlusions meant the disappearance of vascular tissue injuries, the resolution of volume overload, the maintenance of blood pressure, and the optimization of pulmonary blood flow. These events have been previously related to the activation of chymase. The normalized hemodynamics and blood pressure resulting from the PDA occlusions resulted in a reduction in chymase activity after the operations. The echocardiographic parameters indicated that the congestion significantly decreased as a result of the surgical treatment. In the present study, the change in chymase activity reflected the echocardiographic congestion, which indicated that chymase activity reflected the effect of the hemodynamic changes with a high sensitivity. The echocardiographic parameters regarding left ventricular congestion commonly decreased soon after the surgery. However, the cardiovascular damage did not immediately disappear. The post-operative individual differences in chymase activity could depend on the degree of tissue damage and the improvement in pathology in each case. The results of this study suggested that chymase activity could be used as a quantitative indicator of the degree of PDA severity and to understand the pathophysiology of PDA.

There were limitations to this study that should be considered. First, the small sample size did not allow for an analysis of the correlations between chymase activity and the echocardiography parameters, or to evaluate them according to the severity of PDA. Second, plasma chymase activity was not compared with another assay method (such as by using tissue samples). Novel chymase activity measurements require further investigations such as plasma and tissues in healthy dogs, in medically treated PDA dogs, and in dogs with other types of CVD.

In the present study, chymase activity was suggested to be important for understanding the changes in hemodynamics observed before and after a PDA operation. The level of chymase activity might be associated with PDA severity, as the same trend of changes was observed with the echocardiography parameters. Therefore, the measurement of plasma chymase activity was indicated to be useful as a novel predictor of understanding the pathophysiology of PDA in veterinary medicine. In the future, chymase activity may decide the medication, regardless of congestion, or decide the change from an ACE inhibitor to an angiotensin receptor blocker. Chymase activity is expected to become a criterion for the diagnosis of a cardiovascular tissue injury and the treatment of CVD in veterinary medicine.

## Figures and Tables

**Table 1:** Echocardiographic parameters measured at time of diagnose (Pre) and at two months after operations (Post). E vel and E/e’ at LVFW significantly decreased. Standard deviation: SD; 95% confidence interval: CI; fraction shortening: FS; early diastolic left ventricular inflow velocity: E vel; diastolic early mitral ring velocity: e’; significance: * *p* < 0.05.

	Pre (Mean ± SD)	Post (Mean ± SD)	*p*-Value	CI
FS%	39.4 ± 11.8	40.8 ± 8.0	0.2522	−2.600 to 10.10
E vel (cm/s)	108.3 ± 27.2	82.9 ± 12.8	0.0024 *	−47.20 to −10.40
E/e’ IVS	12.2 ± 3.8	9.7 ±2.1	0.0984	−5.150 to 0.6900
E/e’ LVFW	10.9 ± 2.9	8.0 ± 1.6	0.0008 *	−4.140 to −1.470

## Data Availability

Not applicable.

## References

[1] Wang Y., Gu Y., Lewis D.F., Alexander J.S., Granger D.N. (2010). Elevated plasma chymotrypsin-like protease (chymase) activity in women with preeclampsia. Hypertens. Pregnancy.

[2] Ahmad S., Ferrario C.M. (2018). Chymase inhibitors for the treatment of cardiac diseases: A patent review (2010–2018). Expert Opin. Ther. Pat..

[3] Ahmad S., Varagic J., VonCannon J.L., Groban L., Collawn J.F., Dell’Italia L.J., Ferrario C.M. (2016). Primacy of cardiac chymase over angiotensin converting enzyme as an angiotensin-(1-12) metabolizing enzyme. Biochem. Biophys. Res. Commun..

[4] Dell’Italia L.J., Collawn J.F., Ferrario C.M. (2018). Multifunctional Role of Chymase in Acute and Chronic Tissue Injury and Remodeling. Circ. Res..

[5] Hsu H.W., Lin T.Y., Liu Y.C., Yeh J.L., Hsu J.H. (2021). Molecular Mechanisms Underlying Remodeling of Ductus Arteriosus: Looking beyond the Prostaglandin Pathway. Int. J. Mol. Sci..

[6] Gillam-Krakauer M., Mahajan K. (2022). Patent Ductus Arteriosus.

[7] Crockett S.L., Berger C.D., Shelton E.L., Reese J. (2019). Molecular and mechanical factors contributing to ductus arteriosus patency and closure. Congenit. Heart Dis..

[8] Ferguson J.M. (2019). Pharmacotherapy for patent ductus arteriosus closure. Congenit. Heart Dis..

[9] Philip R., Lamba V., Talati A., Sathanandam S. (2020). Pulmonary Hypertension with Prolonged Patency of the Ductus Arteriosus in Preterm Infants. Children.

[10] Zaidi M., Sorathia N., Abbasi H., Khashkhusha A., Harky A. (2020). Interventions on patent ductus arteriosus and its impact on congenital heart disease. Cardiol. Young.

[11] Bonagura J.D., Lehmkuhl L.B., Fox P.R., Sisson D., Moise N.S. (1999). Congenital Heart Disease. Textbook of Canine and Feline Cardiology: Principles and Clinical Practice.

[12] Broaddus K., Tillson M. (2010). Patent ductus arteriosus in dogs. Compend. Contin. Educ. Vet..

[13] Buchanan J.W. (2001). Patent ductus arteriousus morphology, pathogenesis, types and treatment. J. Vet. Cardiol..

[14] Strickland K., Tilley L.P., Smith F.W.K., Oyama M.A., Sleeper M.M. (2008). Congenital Heart Disease. Manual of Canine and Feline Cardiology.

[15] Boon J., Boon J. (2011). The M-mode and Doppler examination. Veterinary Echocardiography.

[16] Hamabe L., Kim S., Yoshiyuki R., Fukayama T., Nakata T.M., Fukushima R., Tanaka R. (2015). Echocardiographic evaluation of myocardial changes observed after closure of patent ductus arteriosus in dogs. J. Vet. Intern. Med..

[17] Ishikawa R., Fujii Y., Takano H., Sunahara H., Aoki T., Wakao Y. (2013). Left ventricular reverse remodeling after ductal closure in dogs with hemodynamically significant patent ductus arteriosus. J. Appl. Res. Vet. Med..

[18] Greet V., Bode E.F., Dukes-McEwan J., Oliveira P., Connolly D.J., Sargent J. (2021). Clinical features and outcome of dogs and cats with bidirectional and continuous right-to-left shunting patent ductus arteriosus. J. Vet. Intern. Med..

[19] Saunders A.B., Gordon S.G., Boggess M.M., Miller M.W. (2014). Long-term outcome in dogs with patent ductus arteriosus: 520 cases (1994–2009). J. Vet. Intern. Med..

[20] Asano K., Kadosawa T., Okumura M., Fujinaga T. (1999). Peri-operative changes in echocardiographic measurements and plasma atrial and brain natriuretic peptide concentrations in 3 dogs with patent ductus arteriosus. J. Vet. Med. Sci..

[21] Weisz D.E., McNamara P.J., El-Khuffash A. (2017). Cardiac biomarkers and haemodynamically significant patent ductus arteriosus in preterm infants. Early Hum. Dev..

[22] Stewart J.A., Wei C.C., Brower G.L., Rynders P.E., Hankes G.H., Dillon A.R., Lucchesi P.A., Janicki J.S., Dell’Italia L.J. (2003). Cardiac mast cell- and chymase-mediated matrix metalloproteinase activity and left ventricular remodeling in mitral regurgitation in the dog. J. Mol. Cell. Cardiol..

[23] Ihara M., Urata H., Shirai K., Ideishi M., Hoshino F., Suzumiya J., Kikuchi M., Arakawa K. (2000). High cardiac angiotensin-II-forming activity in infarcted and non-infarcted human myocardium. Cardiology.

[24] Okamura K., Okuda T., Shirai K., Urata H. (2018). Positive correlation between blood pressure or heart rate and chymase-dependent angiotensin II-forming activity in circulating mononuclear leukocytes measured by new ELISA. Clin. Exp. Hypertens..

[25] Takai S., Jin D., Muramatsu M., Okamoto Y., Miyazaki M. (2004). Therapeutic applications of chymase inhibitors in cardiovascular diseases and fibrosis. Eur. J. Pharmacol..

[26] Caughey G.H., Raymond W.W., Wolters P.J. (2000). Angiotensin II generation by mast cell alpha- and beta-chymases. Biochim. Biophys. Acta.

[27] Parkerson S., Philip R., Talati A., Sathanandam S. (2020). Management of Patent Ductus Arteriosus in Premature Infants in 2020. Front. Pediatr..

